# 1,1′-[4-(2,4-Dichloro­phen­yl)-2,6-di­methyl-1,4-di­hydro­pyridine-3,5-di­yl]diethanone

**DOI:** 10.1107/S1600536810052839

**Published:** 2011-01-15

**Authors:** J. Kalyana Sundar, B. Palakshi Reddy, V. Vijayakumar, S. Natarajan, J. Suresh, P. L. Nilantha Lakshman

**Affiliations:** a Department of Physics, Madurai Kamaraj University, Madurai 625 021, India; b Organic Chemistry Division, School of Science and Humanities, VIT University, Vellore 632 014, India; c Department of Physics, The Madura College, Madurai 625 011, India; d Department of Food Science and Technology, University of Ruhuna, Mapalana, Kamburupitiya 81100, Sri Lanka

## Abstract

In the title compound, C_17_H_17_Cl_2_NO_2_, the central 1,4-dihydro­pyridine ring adopts a flattened-boat conformation. The ethanone substituents of the dihydro­pyridine ring at positions 3 and 5 have synperiplanar (*cis*) or anti­periplanar (*trans*) conformations with respect to the adjacent C=C bonds in the dihydro­pyridine ring. The 2,4-dichloro­phenyl ring is almost planar [r.m.s. deviation = 0.0045 (1) Å] and almost perpendicular [89.27 (3)°] to the mean plane of the dihydro­pyridine ring. In the crystal, an N—H⋯O hydrogen bond links mol­ecules into a zigzag chain along the *ac* diagonal. C—H⋯Cl contacts form centrosymmetric dimers and additional weak C—H⋯O contacts further consolidate the packing.

## Related literature

For background to the pharmaceutical applications of 1,4-dihydro­pyridine derivatives, see: Rose (1989 ▶, 1990 ▶); Salehi & Guo (2004 ▶). For structure–activity relationships among 1,4-dihydro­pyridines, see: Triggle *et al.* (1980 ▶); Janis & Triggle (1984 ▶); Langs & Triggle (1985 ▶).
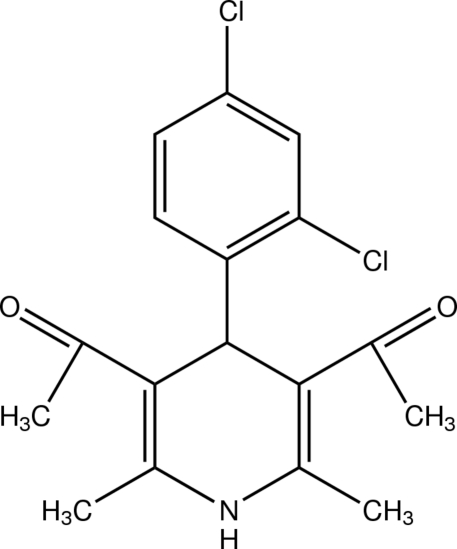


## Experimental

### 

#### Crystal data

C_17_H_17_Cl_2_NO_2_
*M* *_r_* = 338.22
Monoclinic, 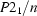

*a* = 10.307 (4) Å
*b* = 13.745 (3) Å
*c* = 11.312 (2) Å
β = 93.80 (2)°
*V* = 1599.0 (8) Å^3^
*Z* = 4
Mo *K*α radiation
μ = 0.41 mm^−1^
*T* = 293 K
0.23 × 0.21 × 0.18 mm

#### Data collection

Nonius MACH3 diffractometer
Absorption correction: ψ scan (North *et al.*, 1968 ▶) *T*_min_ = 0.910, *T*_max_ = 0.929
3247 measured reflections
2811 independent reflections
2265 reflections with *I* > 2σ(*I*)
*R*_int_ = 0.014
3 standard reflections every 60 min  intensity decay: none

#### Refinement

*R*[*F* ^2^ > 2σ(*F* ^2^)] = 0.032
*wR*(*F* ^2^) = 0.096
*S* = 1.05
2811 reflections
207 parameters
H atoms treated by a mixture of independent and constrained refinement
Δρ_max_ = 0.21 e Å^−3^
Δρ_min_ = −0.21 e Å^−3^

### 

Data collection: *CAD-4 EXPRESS* (Enraf–Nonius, 1994 ▶); cell refinement: *CAD-4 EXPRESS*; data reduction: *XCAD4* (Harms & Wocadlo, 1996 ▶); program(s) used to solve structure: *SHELXS97* (Sheldrick, 2008 ▶); program(s) used to refine structure: *SHELXL97* (Sheldrick, 2008 ▶); molecular graphics: *PLATON* (Spek, 2009 ▶); software used to prepare material for publication: *SHELXL97*.

## Supplementary Material

Crystal structure: contains datablocks global, I. DOI: 10.1107/S1600536810052839/sj5076sup1.cif

Structure factors: contains datablocks I. DOI: 10.1107/S1600536810052839/sj5076Isup2.hkl

Additional supplementary materials:  crystallographic information; 3D view; checkCIF report

## Figures and Tables

**Table 1:** Hydrogen-bond geometry (Å, °)

*D*—H⋯*A*	*D*—H	H⋯*A*	*D*⋯*A*	*D*—H⋯*A*
N1—H1⋯O1^i^	0.81 (2)	2.16 (2)	2.951 (2)	163 (2)
C8—H8*B*⋯O2^ii^	0.96	2.56	3.397 (3)	147
C10—H10*C*⋯O2^ii^	0.96	2.43	3.341 (3)	158
C17—H17⋯Cl2^iii^	0.93 (1)	2.92 (1)	3.796 (2)	158 (1)

Symmetry codes: (i) 
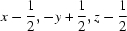
; (ii) 
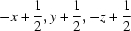
; (iii) 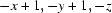.

**Fig. 1.**
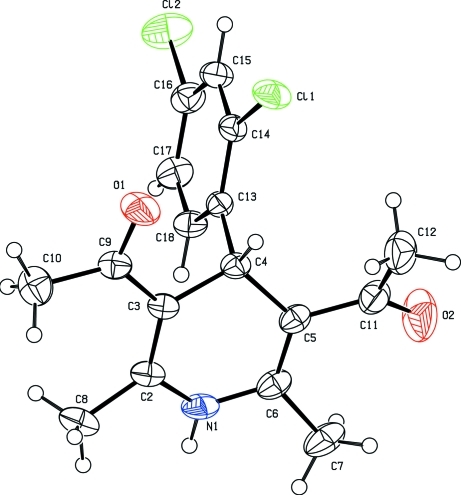
The molecular structure of (I), showing 40% probability displacement ellipsoids and the atom-numbering scheme.

**Fig. 2.**
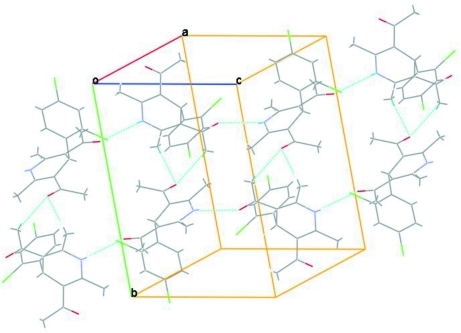
Packing of the crystal structre (I), viewed down the a axis. Hydrogen bonds are shown as dashed lines.

## supplementary crystallographic information

### Comment

1,4-Dihydropyridine derivatives have yielded many drugs which act as calcium channel agonists or antagonists (Rose, 1989, 1990) and various bioactive compounds such as vasodilator, antiatherosclerotic, antitumor, geroprotective, heptaprotective and antidiabetic agents (Salehi & Guo, 2004). Triggle and co-workers (Triggle *et al.*, 1980; Janis & Triggle, 1984; Langs & Triggle, 1985) have identified some important structural requirements for biological activity. We have studied the crystal structure of 1,1'-[4-(2,4-dichlorophenyl)-2,6-dimethyl-1,4-dihydropyridine- 3,5-diyl]diethanone.

In the title compound (I)(Fig. 1), C_17_H_17_Cl_2_NO_2_, the central 1, 4-dihydropyridine ring adopts a flattened boat conformation. The ethanone substituents of the dihydropyridine ring at positions 3 and 5 have different (*cis*/*trans*) configurations with respect to the double bonds in the pyridine ring. Each group is oriented in a synperiplanar (*cis*) or antiperiplanar (*trans*) conformation with respect to the adjacent C═ C in the dihydropyridine ring, which is evident from the torsion angles of C6—C5—C11—O2 [31.44 (40)°] and C2—C3—C9—O1 [172.43 (20)°], respectively.

The methyl groups attached at C2 and C6 positions of the pyridine ring adopt equatorial orientation as can be seen from the torsion angles [C7—C6—N1—C2] 165.54 (20)° and [C8—C2—N1—C6] -164.62 (20)°. The 2,4-dichlorophenyl ring is planar and almost perpendicular to the mean plane of the dihydropyridine ring with the plane angle: 89.27 (3)°. This close to perpendicular orientation of the dichlorophenyl ring to the dihydropyridine ring can be ascribed to the greater steric hinderance with the two ethanone groups at C3 and C5. Atom N1(*x*,*y*,*z*) of the pyridine ring make a intermolecular hydrogen bond with the atom O1(-1/2 + *x*, 1/2 + *y*, -1/2 + *z*), leading to a zigzag chain running along the diagonal of the *ac* - plane (Fig. 2). C17—H17···Cl2 contacts form centrosymmetric dimers and additional weak C—H···O contacts further stabilise the structure, Table 1.

### Experimental

2,4,dicholrobenzaldehyde (10 mmol), acetylacetone (20 mmol) and ammonium acetate (10 mmol) in ethanol were heated on a steam bath until the color of the solution changed to reddish-orange. The mixture was cooled in ice to yield a solid product, which was extracted using diethylether. The purity of the crude product was checked through TLC and recrystallized from acetone/ether 1:1 [yield: 60%, m.p. 218–220°C].

### Refinement

H atoms were placed at calculated positions and allowed to ride on their carrier atoms with C—H = 0.93–0.97 Å, and *U*_iso_ = 1.2*U*_eq_(C) for CH_2_ and CH groups and *U*_iso_ = 1.5*U*_eq_(C) for CH_3_ group. The N-bound H atom is located in a difference Fourier map and its positional parameters were refined.

### Crystal data

**Table:**

C_17_H_17_Cl_2_NO_2_	*F*(000) = 704
*M**_r_* = 338.22	*D*_x_ = 1.405 Mg m^−^^3^
Monoclinic, *P*2_1_/*n*	Mo *K*α radiation, λ = 0.71073 Å
Hall symbol: -P 2yn	Cell parameters from 25 reflections
*a* = 10.307 (4) Å	θ = 2–25°
*b* = 13.745 (3) Å	µ = 0.41 mm^−^^1^
*c* = 11.312 (2) Å	*T* = 293 K
β = 93.80 (2)°	Block, colourless
*V* = 1599.0 (8) Å^3^	0.23 × 0.21 × 0.18 mm
*Z* = 4	

### Data collection

**Table:**

Nonius MACH3 diffractometer	2265 reflections with *I* > 2σ(*I*)
Radiation source: fine-focus sealed tube	*R*_int_ = 0.014
graphite	θ_max_ = 25.0°, θ_min_ = 2.3°
ω–2θ scans	*h* = 0→12
Absorption correction: ψ scan (North *et al.*, 1968)	*k* = −1→16
*T*_min_ = 0.910, *T*_max_ = 0.929	*l* = −13→13
3247 measured reflections	3 standard reflections every 60 min
2811 independent reflections	intensity decay: none

### Refinement

**Table:**

Refinement on *F*^2^	Primary atom site location: structure-invariant direct methods
Least-squares matrix: full	Secondary atom site location: difference Fourier map
*R*[*F*^2^ > 2σ(*F*^2^)] = 0.032	Hydrogen site location: inferred from neighbouring sites
*wR*(*F*^2^) = 0.096	H atoms treated by a mixture of independent and constrained refinement
*S* = 1.05	*w* = 1/[σ^2^(*F*_o_^2^) + (0.0451*P*)^2^ + 0.6776*P*] where *P* = (*F*_o_^2^ + 2*F*_c_^2^)/3
2811 reflections	(Δ/σ)_max_ < 0.001
207 parameters	Δρ_max_ = 0.21 e Å^−^^3^
0 restraints	Δρ_min_ = −0.21 e Å^−^^3^

### Special details

**Table:**

Geometry. All e.s.d.'s (except the e.s.d. in the dihedral angle between two l.s. planes) are estimated using the full covariance matrix. The cell e.s.d.'s are taken into account individually in the estimation of e.s.d.'s in distances, angles and torsion angles; correlations between e.s.d.'s in cell parameters are only used when they are defined by crystal symmetry. An approximate (isotropic) treatment of cell e.s.d.'s is used for estimating e.s.d.'s involving l.s. planes.
Refinement. Refinement of *F*^2^ against ALL reflections. The weighted *R*-factor *wR* and goodness of fit *S* are based on *F*^2^, conventional *R*-factors *R* are based on *F*, with *F* set to zero for negative *F*^2^. The threshold expression of *F*^2^ > σ(*F*^2^) is used only for calculating *R*-factors(gt) *etc*. and is not relevant to the choice of reflections for refinement. *R*-factors based on *F*^2^ are statistically about twice as large as those based on *F*, and *R*- factors based on ALL data will be even larger.

### Fractional atomic coordinates and isotropic or equivalent isotropic displacement parameters (Å^2^)

**Table:**

	*x*	*y*	*z*	*U*_iso_*/*U*_eq_	
C2	0.09331 (17)	0.23909 (14)	0.35680 (16)	0.0368 (4)	
C3	0.21210 (16)	0.23609 (13)	0.41766 (15)	0.0333 (4)	
C4	0.32545 (16)	0.18638 (13)	0.36213 (15)	0.0325 (4)	
H4	0.3770	0.1517	0.4247	0.039*	
C5	0.27979 (18)	0.11313 (13)	0.26722 (15)	0.0371 (4)	
C6	0.16077 (19)	0.12296 (14)	0.21010 (16)	0.0405 (4)	
C7	0.1058 (2)	0.06292 (16)	0.1077 (2)	0.0573 (6)	
H7A	0.0130	0.0706	0.0998	0.069*	
H7B	0.1267	−0.0043	0.1216	0.069*	
H7C	0.1424	0.0841	0.0362	0.069*	
C8	−0.02898 (18)	0.28757 (18)	0.3917 (2)	0.0536 (6)	
H8A	−0.0961	0.2797	0.3295	0.064*	
H8B	−0.0128	0.3556	0.4050	0.064*	
H8C	−0.0562	0.2584	0.4631	0.064*	
C9	0.24581 (18)	0.28036 (14)	0.53385 (16)	0.0388 (4)	
C10	0.1592 (2)	0.34902 (17)	0.59460 (18)	0.0538 (6)	
H10A	0.2028	0.3711	0.6673	0.065*	
H10B	0.0803	0.3162	0.6114	0.065*	
H10C	0.1390	0.4038	0.5440	0.065*	
C11	0.3678 (2)	0.03428 (15)	0.23582 (19)	0.0485 (5)	
C12	0.4571 (3)	−0.00851 (18)	0.3312 (2)	0.0695 (7)	
H12A	0.4768	−0.0745	0.3114	0.083*	
H12B	0.4159	−0.0072	0.4049	0.083*	
H12C	0.5360	0.0287	0.3389	0.083*	
C13	0.41271 (16)	0.26267 (13)	0.30834 (15)	0.0313 (4)	
C14	0.54362 (16)	0.27779 (13)	0.34248 (15)	0.0348 (4)	
C15	0.61739 (18)	0.34868 (15)	0.29079 (17)	0.0422 (5)	
H15	0.7041	0.3580	0.3166	0.051*	
C16	0.56114 (19)	0.40453 (15)	0.20163 (17)	0.0446 (5)	
C17	0.4322 (2)	0.39221 (15)	0.16311 (17)	0.0457 (5)	
H17	0.3943	0.4302	0.1022	0.055*	
C18	0.36099 (18)	0.32216 (14)	0.21721 (16)	0.0394 (4)	
H18	0.2740	0.3142	0.1916	0.047*	
Cl1	0.62411 (4)	0.20669 (4)	0.45204 (5)	0.05059 (17)	
Cl2	0.65383 (6)	0.49418 (5)	0.13777 (6)	0.0727 (2)	
N1	0.07592 (16)	0.19087 (13)	0.25047 (15)	0.0438 (4)	
O1	0.35315 (14)	0.26245 (14)	0.58271 (14)	0.0638 (5)	
O2	0.3688 (2)	0.00154 (15)	0.13564 (16)	0.0885 (7)	
H1	0.006 (2)	0.1959 (17)	0.213 (2)	0.053 (7)*	

### Atomic displacement parameters (Å^2^)

**Table:**

	*U*^11^	*U*^22^	*U*^33^	*U*^12^	*U*^13^	*U*^23^
C2	0.0286 (9)	0.0424 (10)	0.0386 (10)	−0.0049 (8)	−0.0028 (7)	0.0092 (8)
C3	0.0286 (9)	0.0395 (10)	0.0314 (9)	−0.0029 (7)	−0.0007 (7)	0.0038 (7)
C4	0.0304 (9)	0.0363 (9)	0.0297 (9)	−0.0005 (7)	−0.0065 (7)	0.0013 (7)
C5	0.0417 (10)	0.0348 (10)	0.0339 (9)	−0.0050 (8)	−0.0049 (8)	0.0011 (8)
C6	0.0483 (11)	0.0366 (10)	0.0351 (10)	−0.0091 (9)	−0.0095 (8)	0.0048 (8)
C7	0.0707 (15)	0.0496 (13)	0.0480 (12)	−0.0153 (11)	−0.0231 (11)	−0.0001 (10)
C8	0.0283 (10)	0.0693 (15)	0.0624 (13)	0.0005 (10)	−0.0016 (9)	0.0081 (11)
C9	0.0347 (10)	0.0472 (11)	0.0345 (9)	−0.0049 (8)	0.0019 (8)	0.0016 (8)
C10	0.0600 (13)	0.0636 (14)	0.0379 (11)	0.0095 (11)	0.0043 (9)	−0.0027 (10)
C11	0.0546 (12)	0.0396 (11)	0.0503 (12)	−0.0021 (9)	−0.0034 (9)	−0.0076 (9)
C12	0.0770 (17)	0.0506 (14)	0.0778 (17)	0.0214 (12)	−0.0178 (14)	−0.0106 (12)
C13	0.0282 (9)	0.0359 (9)	0.0295 (8)	0.0005 (7)	−0.0009 (7)	−0.0063 (7)
C14	0.0294 (9)	0.0392 (10)	0.0351 (9)	0.0036 (7)	−0.0025 (7)	−0.0043 (8)
C15	0.0290 (9)	0.0505 (12)	0.0469 (11)	−0.0040 (8)	0.0010 (8)	−0.0042 (9)
C16	0.0454 (11)	0.0459 (11)	0.0431 (11)	−0.0097 (9)	0.0076 (9)	−0.0009 (9)
C17	0.0492 (11)	0.0492 (12)	0.0377 (10)	−0.0045 (9)	−0.0051 (9)	0.0058 (9)
C18	0.0333 (9)	0.0474 (11)	0.0365 (10)	−0.0038 (8)	−0.0060 (8)	−0.0003 (8)
Cl1	0.0328 (3)	0.0580 (3)	0.0589 (3)	0.0034 (2)	−0.0119 (2)	0.0099 (2)
Cl2	0.0696 (4)	0.0779 (5)	0.0706 (4)	−0.0307 (3)	0.0044 (3)	0.0198 (3)
N1	0.0329 (9)	0.0529 (10)	0.0431 (9)	−0.0060 (7)	−0.0163 (7)	0.0043 (8)
O1	0.0408 (8)	0.0997 (13)	0.0485 (9)	0.0097 (8)	−0.0152 (7)	−0.0258 (9)
O2	0.1091 (16)	0.0922 (15)	0.0621 (11)	0.0329 (12)	−0.0095 (10)	−0.0350 (10)

### Geometric parameters (Å, °)

**Table:**

C2—C3	1.365 (2)	C10—H10A	0.9600
C2—N1	1.375 (3)	C10—H10B	0.9600
C2—C8	1.502 (3)	C10—H10C	0.9600
C3—C9	1.469 (3)	C11—O2	1.220 (3)
C3—C4	1.524 (2)	C11—C12	1.492 (3)
C4—C5	1.524 (2)	C12—H12A	0.9600
C4—C13	1.533 (2)	C12—H12B	0.9600
C4—H4	0.9800	C12—H12C	0.9600
C5—C6	1.355 (3)	C13—C18	1.394 (3)
C5—C11	1.472 (3)	C13—C14	1.394 (2)
C6—N1	1.378 (3)	C14—C15	1.389 (3)
C6—C7	1.503 (3)	C14—Cl1	1.7444 (18)
C7—H7A	0.9600	C15—C16	1.366 (3)
C7—H7B	0.9600	C15—H15	0.9300
C7—H7C	0.9600	C16—C17	1.382 (3)
C8—H8A	0.9600	C16—Cl2	1.745 (2)
C8—H8B	0.9600	C17—C18	1.378 (3)
C8—H8C	0.9600	C17—H17	0.9300
C9—O1	1.228 (2)	C18—H18	0.9300
C9—C10	1.497 (3)	N1—H1	0.81 (2)
			
C3—C2—N1	119.17 (17)	H10A—C10—H10B	109.5
C3—C2—C8	128.40 (18)	C9—C10—H10C	109.5
N1—C2—C8	112.41 (16)	H10A—C10—H10C	109.5
C2—C3—C9	126.14 (17)	H10B—C10—H10C	109.5
C2—C3—C4	119.48 (16)	O2—C11—C5	122.6 (2)
C9—C3—C4	114.33 (15)	O2—C11—C12	118.9 (2)
C5—C4—C3	112.16 (14)	C5—C11—C12	118.43 (18)
C5—C4—C13	109.52 (14)	C11—C12—H12A	109.5
C3—C4—C13	110.04 (14)	C11—C12—H12B	109.5
C5—C4—H4	108.3	H12A—C12—H12B	109.5
C3—C4—H4	108.3	C11—C12—H12C	109.5
C13—C4—H4	108.3	H12A—C12—H12C	109.5
C6—C5—C11	120.76 (17)	H12B—C12—H12C	109.5
C6—C5—C4	119.86 (17)	C18—C13—C14	115.67 (16)
C11—C5—C4	119.35 (16)	C18—C13—C4	119.28 (15)
C5—C6—N1	118.96 (17)	C14—C13—C4	125.05 (16)
C5—C6—C7	126.61 (19)	C15—C14—C13	122.33 (17)
N1—C6—C7	114.35 (18)	C15—C14—Cl1	116.38 (13)
C6—C7—H7A	109.5	C13—C14—Cl1	121.28 (14)
C6—C7—H7B	109.5	C16—C15—C14	119.20 (17)
H7A—C7—H7B	109.5	C16—C15—H15	120.4
C6—C7—H7C	109.5	C14—C15—H15	120.4
H7A—C7—H7C	109.5	C15—C16—C17	121.10 (18)
H7B—C7—H7C	109.5	C15—C16—Cl2	119.04 (15)
C2—C8—H8A	109.5	C17—C16—Cl2	119.85 (16)
C2—C8—H8B	109.5	C18—C17—C16	118.35 (18)
H8A—C8—H8B	109.5	C18—C17—H17	120.8
C2—C8—H8C	109.5	C16—C17—H17	120.8
H8A—C8—H8C	109.5	C17—C18—C13	123.34 (17)
H8B—C8—H8C	109.5	C17—C18—H18	118.3
O1—C9—C3	118.17 (17)	C13—C18—H18	118.3
O1—C9—C10	117.81 (17)	C2—N1—C6	124.61 (16)
C3—C9—C10	123.98 (17)	C2—N1—H1	118.2 (16)
C9—C10—H10A	109.5	C6—N1—H1	116.5 (16)
C9—C10—H10B	109.5		
			
N1—C2—C3—C9	−177.66 (17)	C4—C5—C11—C12	34.8 (3)
C8—C2—C3—C9	0.5 (3)	C5—C4—C13—C18	62.3 (2)
N1—C2—C3—C4	4.8 (3)	C3—C4—C13—C18	−61.4 (2)
C8—C2—C3—C4	−177.01 (18)	C5—C4—C13—C14	−116.97 (18)
C2—C3—C4—C5	−22.3 (2)	C3—C4—C13—C14	119.30 (18)
C9—C3—C4—C5	159.92 (15)	C18—C13—C14—C15	1.2 (3)
C2—C3—C4—C13	99.89 (18)	C4—C13—C14—C15	−179.54 (16)
C9—C3—C4—C13	−77.89 (18)	C18—C13—C14—Cl1	−177.96 (13)
C3—C4—C5—C6	24.6 (2)	C4—C13—C14—Cl1	1.3 (2)
C13—C4—C5—C6	−97.9 (2)	C13—C14—C15—C16	−1.4 (3)
C3—C4—C5—C11	−157.55 (16)	Cl1—C14—C15—C16	177.78 (15)
C13—C4—C5—C11	80.0 (2)	C14—C15—C16—C17	0.6 (3)
C11—C5—C6—N1	173.01 (18)	C14—C15—C16—Cl2	179.48 (15)
C4—C5—C6—N1	−9.1 (3)	C15—C16—C17—C18	0.2 (3)
C11—C5—C6—C7	−3.6 (3)	Cl2—C16—C17—C18	−178.61 (16)
C4—C5—C6—C7	174.23 (18)	C16—C17—C18—C13	−0.4 (3)
C2—C3—C9—O1	172.37 (19)	C14—C13—C18—C17	−0.3 (3)
C4—C3—C9—O1	−10.0 (3)	C4—C13—C18—C17	−179.59 (18)
C2—C3—C9—C10	−10.0 (3)	C3—C2—N1—C6	13.8 (3)
C4—C3—C9—C10	167.64 (18)	C8—C2—N1—C6	−164.67 (18)
C6—C5—C11—O2	31.2 (3)	C5—C6—N1—C2	−11.5 (3)
C4—C5—C11—O2	−146.6 (2)	C7—C6—N1—C2	165.50 (18)
C6—C5—C11—C12	−147.4 (2)		

### Hydrogen-bond geometry (Å, °)

**Table:**

*D*—H···*A*	*D*—H	H···*A*	*D*···*A*	*D*—H···*A*
N1—H1···O1^i^	0.81 (2)	2.16 (2)	2.951 (2)	163 (2)
C8—H8B···O2^ii^	0.96	2.56	3.397 (3)	147
C10—H10C···O2^ii^	0.96	2.43	3.341 (3)	158
C4—H4···Cl1	0.98	2.65	3.190 (2)	115
C17—H17···Cl2^iii^	0.93 (1)	2.92 (1)	3.796 (2)	158.(1)

Symmetry codes: (i) *x*−1/2, −*y*+1/2, *z*−1/2; (ii) −*x*+1/2, *y*+1/2, −*z*+1/2; (iii) −*x*+1, −*y*+1, −*z*.

## References

[bb1] Enraf–Nonius (1994). *CAD-4 EXPRES*S. Enraf–Nonius, Delft, The Netherlands.

[bb2] Harms, K. & Wocadlo, S. (1996). *XCAD4* University of Marburg, Germany.

[bb3] Janis, R. A. & Triggle, D. J. (1984). *Drug Dev. Res.* **4**, 257–274.

[bb4] Langs, D. A. & Triggle, D. J. (1985). *Mol. Pharmacol.* **27**, 544–548. 2581123

[bb5] North, A. C. T., Phillips, D. C. & Mathews, F. S. (1968). *Acta Cryst.* A**24**, 351–359.

[bb6] Rose, U. (1989). *Arzneim. Forsch. (Drug Res.)*, **39**, 1393–1398.

[bb7] Rose, U. (1990). *Arch. Pharm. (Weinheim)*, **323**, 281–286. 10.1002/ardp.19903230506 2166493

[bb8] Salehi, H. & Guo, Q. X. (2004). *Synth. Commun.* **34**, 4349–4357.

[bb9] Sheldrick, G. M. (2008). *Acta Cryst.* A**64**, 112–122. 10.1107/S0108767307043930 18156677

[bb10] Spek, A. L. (2009). *Acta Cryst.* D**65**, 148–155. 10.1107/S090744490804362X PMC2631630 19171970

[bb11] Triggle, A. M., Shefter, E. & Triggle, D. J. (1980). *J. Med. Chem.* **23**, 1442–1445. 10.1021/jm00186a029 6256552

